# Exploration and practice of humanistic education for medical students based on volunteerism

**DOI:** 10.1080/10872981.2023.2182691

**Published:** 2023-02-25

**Authors:** Lizhi Chen, Jiayi Zhang, Yingjun Zhu, Jie Shan, Luxian Zeng

**Affiliations:** a Department of Science and Education, Guangdong Second Provincial General Hospital, Guangzhou, P.R. China; b School of Medicine, Jinan University, Guangzhou, P.R. China; c Unions of Trade, Guangdong Second Provincial General Hospital, Guangzhou, P.R. China

**Keywords:** Medical student, humanistic education, humanities, volunteerism, practice

## Abstract

Humanistic education aims to promote educated people’s practical and conscious activities to enhance their humanity, cultivate ideal personalities, and realize personal and social values, to develop a humanistic spirit. The advancement of higher education in China has led to the proposal to strengthen scientific and humanistic education integration. Medicine is between science and humanities, shouldering the important task of training senior medical personnel, the quality of medical students will affect the quality of future medical and health work; thus, medical students must explore and practice humanistic education. Promoting and practicing volunteerism is a specific act of constructing spiritual civilization in the whole society, and it is also considered beneficial for improving citizens’ sense of responsibility and dedication. Medical students’ practice of volunteerism and help in society is a precise manifestation of humanistic care. This review summarizes medical students’ exploration and practice of humanistic education in volunteering.

## Background

The future career path of medical students entails bearing the significant responsibility of saving lives and treating patients. Medical humanities are receiving increased attention as part of the current medical education due to their inclusion in the curriculum, promoting the development of empathetic, compassionate, and culturally sensitive physicians [1–3]. Volunteerism has recently become highly valued, and its guiding principle of ‘dedication, friendship, mutual help, and progress’ is a powerful tool for personal improvement, teamwork, and career development [4,5]. Through volunteer service, we explore the area of humanistic education combined with medical students’ clinical teaching. We hope to guide them in establishing a long-term volunteer service mechanism by conducting such service with the characteristics of medical professionals to educate medical students through ‘practice’ to promote the establishment of good character and values, increase the sense of social responsibility, and help them grow into qualified and competent medical talents.

## Current status of humanistic education

### Basic connotation of humanistic education

The basic meaning of humanistic education is to pass on values rich in humanism and humanistic qualities through teaching by example after paying attention to the meaning of life and its values. Such education includes humanistic behavior, spirit, and knowledge which are integrated and complement each other. Humanistic behavior is the outward expression of humanistic spirit and knowledge usually exhibited as social behavior, which lies in the care and reverence for the life of all things. It also includes the pursuit and aspiration for value and meaning, promoting the harmonious unity of man and man, man and nature, man and society, and gaining the noble benefits of self-pleasure and self-redemption. The humanistic spirit guides the moral level of humanistic education and its system, which focuses on the expression of the humanities and is concerned with human values and spiritual expression. Medical societies prepare students to deal with complex clinical problems by developing critical thinking and understanding personal values, empathy, cultural competence, leadership, and teamwork [6,7]. Humanistic knowledge is an essential component of humanistic education, and only a solid foundation of knowledge allows such education to be put into practice rather than being reduced to empty talk.

### Problems of humanistic education

The traditional medical education model prioritizes the acquisition of professional knowledge and clinical skills, while the importance of humanistic values and the cultivation of medical ethics is frequently overlooked [8]. The traditional biomedical model is shifting to ‘bio-psycho-social medicine.’ The consensus on collaborative medical education reform reflects the significance and ‘unstoppability’ of humanistic medical education. The main current trends are integrating humanistic education into medical education, cultivating medical talents with a humanistic spirit, and strengthening the integration of humanistic and medical science spirits [9]. Primary medical schools begin to pay attention to humanistic education; however, because such education is in its infancy, many problems and dilemmas remain to be resolved.

#### A single teaching format of medical humanistic education

Most medical schools currently offer humanistic education courses in traditional classroom settings, with theoretical examinations serving as the sole assessment criteria [10,11]. While this format allows students to become acquainted with and develop their theoretical knowledge of humanistic education, it also makes it difficult for them to apply what they learn in the classroom to real life. It falls into the category of valuing only humanistic knowledge and spirit while ignoring humanistic behavior. Humanistic education can foster humanistic attitudes, but it does not guarantee that students’ behavior will change [12]. For a long time, medical students have had a superficial understanding of the theoretical knowledge of humanistic education, but they could not derive satisfaction and self-pleasure from it, which greatly reduces their motivation and interest in learning [13,14].

**Figure 1.**
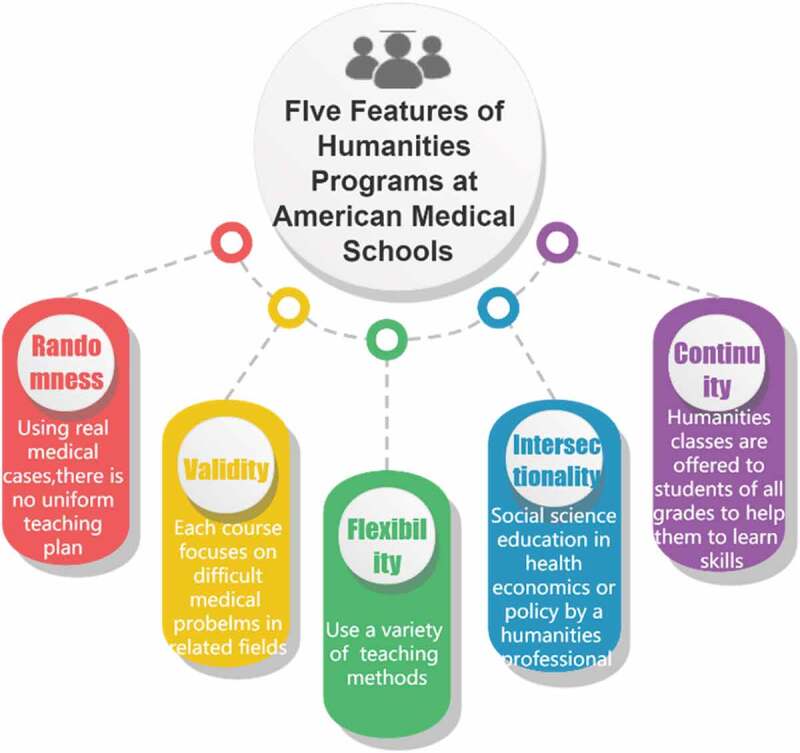
Five features of the humanities program at American medical schools.

Real, independent, participatory experiences and opportunities to address issues that arise during medical humanistic education can better contribute to medical students’ understanding of medical humanities and thus to the development of medical humanistic education [15]. This is why teaching in a way that allows students to feel more involved has greater value. Many teaching methods are included in the University of California, San Francisco’s humanistic education program that is worth considering. Students can participate in a humanistic book club for a spiritual feast, a medical humanistic interest group for mutual self-improvement, and multiple peer-group seminars for independent or supervised learning. Overall, the humanistic curriculum in U.S. medical schools has five characteristics (Figure 1): randomness, effectiveness, flexibility, intersectionality, and continuity [11]. In China, current medical students do not lack a sense of morality, ethical awareness, and responsibility; they lack basic humanistic competencies such as empathy, compassion, and communication skills and should therefore receive complimentary humanistic education. At the national level, there is a need to break away from traditional teaching methods and adopt novel ones to improve their effectiveness [16,17].

Previous research has revealed that implementing a new ‘patient-as-teacher’ approach in medical schools can impact medical students [18]. Students are exposed to the experiences of illness and the health care system by having patients share their stories with them as teachers. Traditional medical education overlooks illness experiences and their impact; discussions between ‘patient teachers’ and medical students can foster a greater sense of medical humanism in medical students, and stories allow students to understand and respond to the suffering of others, develop empathy, and begin to view their medical practice more humanely [19]. The ‘patient-as-teacher’ approach to humanistic education emphasizes the patient’s humanity and the physician’s social and humanistic role in providing patient-centered care, and this approach is worth studying in medical schools.

#### The lag and uncertainty of medical humanistic education

Different societies and economies exist in different times, and the people living in them have different habits and changing lifestyles that together make up the humanities. Therefore, humanities are a product of society and economy, and it often appears after the development of society and economy. Humanistic education also comes from the humanities, so it tends to lag behind and does not be ahead of its time. Moreover, humanistic education is different from general medical education which is based on complex clinical tests. Today’s medicine is in a highly developed stage, and the content of general medical education is less changed, so the development of humanistic education inevitably lags behind compared to general education. Although medical education reform has recently emphasized medical humanities to improve the professionalism of future doctors, the pace of integration of medical humanities in China has lagged behind that of Western countries due to arbitrary medical humanities courses, lack of organizational independence, and insufficient faculty. The medical humanistic education courses offered by medical schools have not met the urgent needs of society [20].

In addition, unlike traditional education, humanistic education is basic education, which includes a wide range of contents, including the relationship between human beings and their own reason, emotion and will, as well as the relationship between human beings and society and nature, and it reflects a person’s ideological and theoretical level and basic aesthetic quality. Different people will have different views on the same event, which makes humanistic education have no ‘standard answer’ that can be applied to any situation. This standard is the value judgment standard provided by moral education for humanistic education, and moral education sets the value orientation of humanities education. Humanistic education is an education in the pursuit of truth, goodness and beauty, and this standard of truth, goodness and beauty is explained by the content of moral education. For any country, moral education is indispensable. Moral education is an organized and planned activity to exert moral influence on educated people in accordance with the requirements of a socially accepted moral code system [21]. The ‘standard answer’ of humanistic education is neither the only one nor the best, but it is only the near-perfect one negotiated over time. Better answers will always appear in the future. Moreover, the teaching of humanistic education can show its uncertainty compared to compulsory medical courses like biochemistry and human anatomy, which have countless experimental data and clinical proof.

#### The general environment of humanistic medical education has not yet been built

Despite the efforts of major medical schools, the construction of the general environment of humanistic medical education has not yet been completed, and many aspects of humanistic medical education still have deficiencies; as a result, such education remains in its early stages. In the bio-psycho-social medicine model era, many foreign medical universities have included humanities in their compulsory curriculum and recognized their value. China has promoted medical humanities, although its completion and implementation lag behind Western countries.

Contemporary Chinese society is facing not only medical problems in the field of biomedical technology, but also how to better promote the integration of medical science and medical humanism, which has become one of the most urgent problems in China. The ‘medical technology comes first’ mentality has led to a technology-oriented medicine that neglects the humanism in medical practice. However, in the era of the biological-psychological-social medicine model, a specialized technical-scientific approach to education is increasingly considered inadequate for the 21st century physician. Although medicine is a science and its purpose is to study diseases, clinicians should always remember that patients are human beings and should consider ‘To Cure Sometimes, To Relieve Often, To Comfort Always’ as the requirements and goals of a qualified doctor, so as to better promote the integration of medical scientific spirit and medical humanism [20]. Moreover, it is more crucial to strengthen the humanistic quality of medical education, cultivate medical talents with a humanistic spirit, and emphasize the humanistic nature of medical education. Other pressing issues in Chinese healthcare include distrust between doctors and patients, violence against medical personnel, commercialization of healthcare, and unethical incentives for medical personnel [22,23]. Nowadays, there are numerous humanistic approaches to medicine, many reviews of the medical humanities, and frequent calls for more emphasis on the humanities in medical education; however, most literature is written in Chinese. Similarly, researchers’ work in China is not systematically presented to and evaluated by an international audience because their work is relatively isolated, and their results are published in disparate journals. All factors mentioned above have influenced and hindered the development of a general environment for humanistic education.

Medicine falls between the sciences and humanities and has scientific and humanistic attributes. Medical students in China must be reminded that the care they provide requires scientific knowledge and medical humanism. Similarly, humans and the environment have a mutually beneficial relationship. Humans create the environment, and the environment influences humans. The inability of medical students to learn medical knowledge in the context of humanistic education leads to a sluggish development of medical humanistic education today. Institutions and forms of humanistic education and settings where humanistic behavior, spirit, and knowledge can be learned and practiced are lacking. If humanism and humanistic qualities are not shown comprehensively to medical students, it will be difficult to build the values of medical students that are perfect and influenced by humanistic education [24].

### The importance of humanistic education in teaching medical students

**Figure 2.**
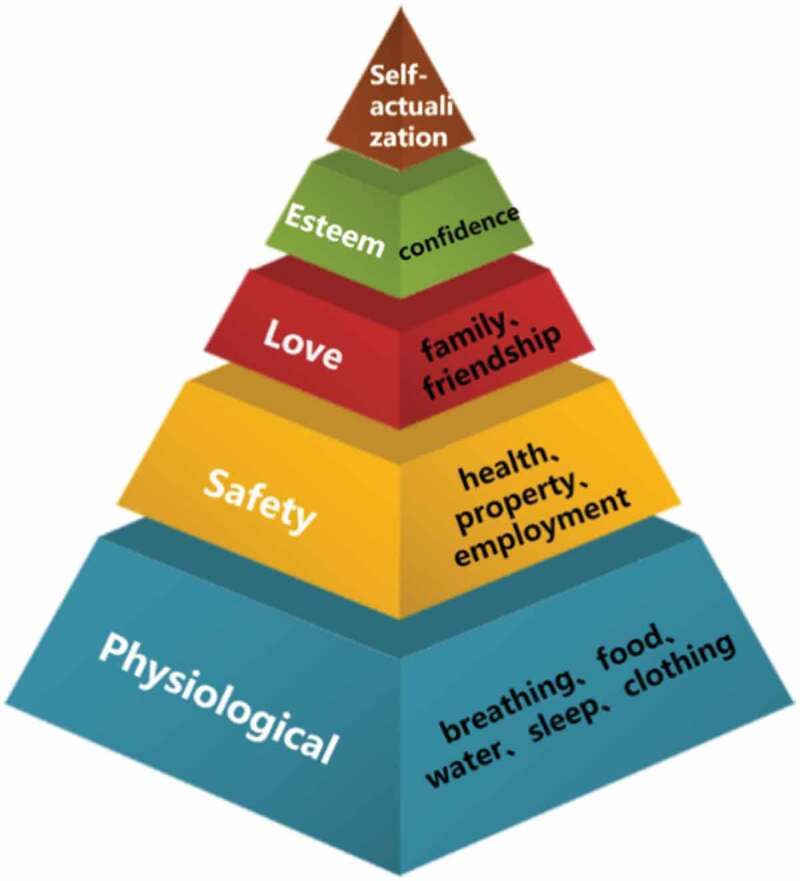
Maslow’s hierarchy of human needs.

In the twenty-first century, healthcare professionals are expected to provide more than just medical care and shift to holistic care. Medicine can be more than just a science; it can help people discover their value in life and achieve self-actualization and self-healing. Maslow’s hierarchy of human needs (Figure 2) divides needs into five categories: physiological, security, belonging, respect, and self-actualization, in descending order; this provides an insightful map for understanding humanistic desires [25,26]. In Watson’s theory of human care, humanistic care is viewed as a fundamental belief, and it is argued that in the process of caring, inner strength allows people to grow and change [27–29]. Thus, professional medical personnel have more duties than merely treating patients, and medical students require qualities other than medical knowledge, such as career-long humanistic values and humanistic care for patients, which cannot be learned in the classroom. This indicates that medical humanities must be added to the medical curriculum to develop medical students’ sensitivity, empathy, and understanding of the human condition [30,31].

The ultimate mission of humanistic medical education is to prepare physicians to educate themselves and have a lifelong commitment to a philosophy of understanding paradoxical coincidences through experience [32,33]. If humanistic medical education is to be promoted and developed, the exploration and practice of such education by medical students should be enhanced. And then they can spiritually develop their own humanity, acquire empathy for patients through self-elevation, and comprehend the nature of mutual healing through the realization of primitive life. Many thoughtful medical leaders are convinced that when physicians are more humane in their interactions with patients, their patients will have more positive health outcomes.

Moreover, doctor-patient relationship has always been one of the hot spots of social concern in China. Although the current status of doctor-patient relationship in China is good overall, there is still local disharmony [34]. The COVID-19 outbreak at the beginning of 2020, as well as the ‘scattered, small outbreaks’ in the post-pandemic era, all point to the increased demand for medical care, the possibility of increased conflicts in doctor-patient relationships. There are many factors that affect the creation of doctor-patient conflicts, including the imperfection of the medical system, the incorrect guidance of social opinion, the asymmetry of information between doctors and patients, and the lack of trust and poor communication between doctors and patients. Implementing humanistic education can well promote the development of doctors’ professional ethics, which can improve the current tense doctor-patient relationship in China to a certain extent and build a harmonious society [35].

## The role of medical students’ participation in volunteer service concerning humanistic education

### Participation in volunteerism is an expression of humanistic behavior

A study investigated the motivations of medical students who participated in volunteer work [36–38]. Most students wanted to make valuable contributions to others and society; some felt it was their responsibility to use their clinical skills, and some expressed other motivations for volunteering, such as gaining more knowledge and needing to be appreciated and affirmed. The motivation of volunteers is often multifaceted, and most medical students choose to participate in volunteer service activities in order to help others and society by showing their own value, which is exactly the manifestation of humanistic care in volunteer service.

The global elderly population is increasing [39], and one of the challenges of aging is to combat loneliness among older adults. Loneliness can have serious health consequences, such as cardiovascular disease and poorer mental health [40]. A study of nursing home residents found an association between emotional loneliness and mortality [41]. Therefore, helping older people overcome loneliness is one of the issues that need to be addressed in today’s society. Research has shown that finding some human resources, such as volunteers, can go some way to helping older adults overcome loneliness. This is because older adults have many unmet needs, volunteers can play an essential role in alleviating their pain and loneliness by providing meaningful dialogue. Volunteer organizations can set up volunteer groups specifically for serving the elderly and plan activities such as cultural performances and senior clubs to help the elderly overcome loneliness in a variety of ways. It implying that volunteers can, to some extent, offer older adults something that healthcare professionals cannot [42]. These are the things volunteers bring to their service to others and society in terms of spiritual solace and humanistic values.

To become an ideal doctor, a medical student must have medical knowledge, a heart for service, good communication skills, and other humanistic aspects of care. It is necessary to provide opportunities for medical students to learn through various activities rather than merely listening to lectures and learning in a classroom [4]. Volunteering allows medical students to improve all aspects of their abilities, including professionalism, empathy, communication skills and teamwork. Furthermore, it practically strengthens medical students’ professional knowledge and allows them to apply clinical skills learned in the classroom.

### Volunteering enriches the humanistic spirit

The humanistic spirit is expressed as the maintenance, pursuit, and concern for human dignity, value, and destiny; a high value of people’s various spiritual and cultural phenomena; and affirmation and molding of an ideal personality with comprehensive development. A literature review reveals that some medical schools instill humanistic values in medical students and foster humanism in medical education through anatomy teaching and donor appreciation ceremonies [8]. However, few medical schools have implemented volunteerism to cultivate humanism among medical students, whose active participation in it can shape the quality of their dedication and establish the value of their service to society. Volunteering allows medical students to consider the medical and macro-social aspects, broadening their horizons. Similarly, volunteerism is generally in demand at the grassroots level, where various disadvantaged groups are located. By reaching such a level and participating in social support and public services, medical students can understand societal needs and gain insight into social trends.

With the growth of health care, hospice care has become a popular area of humanistic medical care. Hospice care is a type of medical care that focuses on alleviating the symptoms of a patient’s illness and slowing its progression in the weeks or even months before their death [43]. Research has shown that medical students can develop personal reflection and empathy skills as hospice volunteers [44]. Empathy is essential in doctor-patient interactions because it improves communication, eases the doctor-patient relationship, and improves medical treatment. It is also central to the humanist ethos [45].

Since the COVID-19 disease swept the world in 2019, a silent but intense war has erupted between humans and the virus [46], with doctors undoubtedly becoming the main frontline force. Medical student volunteers, who were not formally qualified as physicians but were equipped with medical knowledge, actively participated in this dangerous war worldwide [47]. A study was conducted to qualitatively and quantitatively examine the psychological burden and experiences of Chinese medical student volunteers after exposure to COVID-19. The study found that medical student volunteers involved in outbreak prevention and control experienced low levels of psychological distress, indicating that the majority were able to maintain a positive mindset and were willing to volunteer as medical assistants when the country needed them out of a sense of responsibility toward their future career [48,49]. While medical students were not required to fight the COVID-19 pandemic, they provided adequate assistance to the health care system in emergencies and will be needed if similar situations arise [50]. The humanism embodied by medical student volunteers in the fight against the COVID-19 pandemic enhanced the sense of identity of socialism with Chinese characteristics and the leadership of the Communist Party of China. It promoted national self-confidence and traditional Chinese virtues.

### Volunteering supplements humanistic knowledge

Volunteering can effectively enrich medical students’ extracurricular time. While professional courses can be tedious, volunteering adds a ‘splash of color’ to medical students’ after-school life, effectively providing variety in their schooling, improving their learning environment. With its rich and flexible forms and the choice of the breadth and depth of independent participation, volunteering activities provide medical students with more possibilities to participate in social activities and also provide an important way to improve their abilities, which include not only general thinking and social skills, but also professional skills and learning abilities, so volunteering can, to a certain extent, improve the efficiency of medical students’ acceptance of professional knowledge. At the same time, volunteering can broaden their horizons, increase their knowledge, and provide an opportunity and platform for them to escape the ‘ivory tower’ and interact with the humanities outside the campus. Research has revealed that medical students who volunteered a great deal were on par academically with those who did not volunteer or volunteered less, indicating that volunteering complements what medical students cannot learn from books but can also be conciliated with the school curriculum [51,52].

After volunteering in medical school, about 80% of students consider it a mandatory part of their college life. This is because for medical students, the ultimate purpose of receiving medical education is to serve patients and benefit the society. For example, volunteering activities such as public welfare teams, community clinics, sending doctors to the countryside, and helping people with love are conducive to enhancing their sense of cultural identity of caring for society and others, their sense of professional mission of revering life and caring for patients, and their sense of urgency to pay attention to the reform of the healthcare service system and the ethical development of life frontier technology. This kind of experience and feeling rooted in their hearts will become the inexhaustible motivation for them to improve their own medical humanistic quality. This undoubtedly promotes the formation of students’ perception of the value of learning in the present, and also points out the direction for students’ choice of specific specialties in the future and their participation after graduation [53]. Furthermore, students can generate reflection and self-reflection when confronted with new problems in the course of volunteering [54,55]. They can also gain new experiences in solving these problems, and students who participate in service learning can improve their interaction with patients and their clinical skills to understand patient health disparities, all of these are beneficial for learning [56,57].

Senior medical students in clinical practice focus more on the patient’s illness, neglecting humanistic care. Volunteering helps them have more opportunities to bond with people and provides a platform to learn about humanities. Junior medical students focus on basic medical knowledge, rarely communicate with people, and lack the opportunity to experience humanistic education. This volunteer service, which accommodates all students regardless of their grades, compensates for their lack of opportunities for human contact.

## Volunteerism in the practice of humanistic education for medical students

### Prerequisites for medical students to experience humanistic education from volunteerism

Studies have found no significant difference in age, gender, race/ethnicity, hometown, and academic achievement among students with similar interest in service, and the level of volunteering may be positively correlated with the level of interest in favor [58]. Therefore, the prerequisite for medical student volunteering is that students choose to do it voluntarily rather than being forced to, which is the organizing principle of volunteering and the fundamental right of medical students as independent members of society. To promote the primary task of subjective motivation, a medical school or volunteer activity organizer must ensure students’ right to be informed and consent and help them deeply understand the meaning of volunteering. Students should not be forced to complete volunteer service directly or indirectly through any promotion conditions, rewards, or punishments, as this will lead to negative attitudes toward volunteering. Furthermore, when students volunteer, they will not perceive the real meaning but will simply do the job to complete the task. This is against the spirit of volunteerism, but it is also detrimental to the development of medical students, who do not benefit from humanistic education.

Volunteering requires a gamut of knowledge and skills. When medical students volunteer, they can discover their deficiencies, stimulating their initiative and motivation to learn while eliminating negative psychological traits such as complacency and arrogance. Similarly, volunteering necessitates a more altruistic outlook; thus, educators in medical schools must focus on cultivating medical students’ motivation to volunteer. Meanwhile, policymakers in governments and social organizations must actively create various service platforms and provide more opportunities for participation to motivate medical students to volunteer [59]. When medical students understand the meaning of volunteerism and the value of ‘dedication, love, mutual help, and progress’ through the guidance of schools or volunteer organizers and choose a suitable volunteer activity according to their own will, they will be enthusiastic about it and dedicate themselves to it. Thus, they will be able to support one another through volunteer activities and give back to society, gaining a subtle but practical understanding of humanistic education [60].

### The significance of humanistic education for medical students based on volunteer practice

Volunteerism brings great economic benefits to a country and warmth, love, and happiness to people [61]. Medical students, in particular, must put their knowledge into practice through volunteerism, and excellent humanistic education is the best way to help them develop this awareness. A study demonstrated the relationships between volunteer work and six aspects of personal well-being: happiness, life satisfaction, self-esteem, sense of control over life, physical health, and depression. The results showed that volunteer work improves all six aspects of well-being [62] and if the relevant departments can further measure the individual personality of different volunteers when arranging volunteer work, so that they can each share their strengths, it will not only improve the work results to a certain extent, but also further enhance the happiness level of each person in volunteer service [63,64]. Therefore, a successful volunteer service brings a certain value to others and society and immeasurable value to one’s life in the process [65].

Furthermore, regardless of the success of the volunteer services involved, medical students can identify the methods and reasons for success and failure to develop strong mental capacity and good mental health, which they will need to adapt to their intense future work. For example, one person cannot do volunteering alone but requires team cooperation. Completing volunteer service often involves collaboration and communication between students, students and leading teachers, and students and community members. When volunteers do their work, they understand team consciousness that is incomparable to written knowledge acquired in the classroom, and the importance of team consciousness in their future medical career is self-evident [66,67]. When doctors can reach good cooperation with nurses and doctors from other departments during consultation, the probability of doctor-patient disputes, misdiagnosis and missed diagnosis will be reduced to a certain extent; in addition, whether doctors from different departments can contribute to emergency care and help each other according to their specialties is also related to the prognosis and life and death of patients to a certain extent [5].

## Discussion

This study clarifies the significance of medical students’ exploration and practice of humanistic education based on volunteerism. We offer solutions to improve medical students’ voluntary service due to their immaturity in exploring and practicing humanistic education.

A review of the related literature reveals that the current volunteering model is still relatively simple, consisting of ‘people receiving help ^_^ volunteer activities ^_^ volunteers.’ As volunteering and humanistic education share the same core content, they cannot be organically integrated with humanistic education and cannot be promoted through relatively mature volunteer activities. Therefore, this study makes the following recommendations to improve the volunteering model so that volunteerism and humanistic education can benefit and develop together:

- Make use of medical students’ professional skills to assist those with specific medical needs, such as terminal cancer patients, their families, and disabled children. A backstage matching mechanism should be used in various volunteer services to prioritize matching activities that correspond to their majors to ensure the use of high-quality resources. Reasonable distribution of labor is needed; before conducting volunteer services, organizers should become acquainted with each student’s personality to assign the most appropriate position based on their skills [68]. It is also necessary to determine the appropriate roles for medical students to be ready, willing, and able to serve when needed, thus laying a solid foundation for their humanistic education [69].
- Make volunteers undergo skill training before performing volunteer service to improve their practical ability and accelerate learning efficiencies while in their positions. This approach, like pre-studying before learning, can lead to accomplishments with half the effort, laying a solid foundation for medical students’ humanistic education.
- More volunteer opportunities can be created by society, and rich content and forms of volunteer activities and a perfect volunteer service system and sufficient infrastructure can be established to provide adequate social support for volunteer activities [53]. Research has shown that volunteers can motivate themselves when they perceive social support, thus promoting volunteer behavior [70].
- The state and government must improve volunteerism and provide safer guarantees for volunteers in serving patients to maximize their potential and minimize their exposure to danger, thereby reducing the factors that may hamper them from participating in such activities due to health concerns [71–73].
- Screening and inviting public figures with good qualities to serve as ambassadors increases social influence and motivates students to thrive under the leadership of role models while encouraging them to have a positive motivation to help and assist them in better understanding volunteerism. This builds a roof over the humanistic education of medical students and determines the new heights that the medical career can reach [74].

## Conclusion

In summary, humanistic education plays a significant role in society and is responsible for consolidating the infrastructure of future social stability and harmony. It not only cultivates good communication skills and enhances empathy among medical students, but also improves the professional ethics of medical personnel, thus solving the problem of doctor-patient tensions that seriously affect social harmony under current medical conditions, resolving conflicts between doctors and patients, and cultivating medical talents with both excellent medical knowledge and solid humanistic qualities. However, the implementation of humanistic education in today’s Chinese education system is still in its early stages, and major medical schools try to integrate medical humanities into traditional medical education in various ways. In this work, we explored a volunteer humanistic education approach that combines volunteerism with clinical teaching for medical students. It is concluded that in volunteering, medical students can not only bring value to others and society but also enhance their well-being to a certain extent, cultivate various abilities, develop personal reflection and empathy skills, and demonstrate humanistic spirit and literacy. Finally, by combining the relevant literature to propose improvements to the volunteer service model of medical students, it is expected that the state, society, and schools will collaborate to create a perfect environment for humanist education, instill the humanist spirit into the heart of every medical student, and scientifically establish a good medical environment.
